# Angiogenesis in male breast cancer

**DOI:** 10.1186/1477-7819-3-16

**Published:** 2005-03-02

**Authors:** Evan M Frangou, Joshua Lawson, Rani Kanthan

**Affiliations:** 1 Department of Pathology, University of Saskatchewan, Royal University Hospital, 103 Hospital Drive, Saskatoon, Canada; 2 Institute of Agricultural Rural Environmental Health, University of Saskatchewan, Royal University Hospital, 103 Hospital Drive, Saskatoon, Canada

## Abstract

**Background:**

Male breast cancer is a rare but aggressive and devastating disease. This disease presents at a later stage and in a more advanced fashion than its female counterpart. The immunophenotype also appears to be distinct when compared to female breast cancer. Angiogenesis plays a permissive role in the development of a solid tumor and provides an avenue for nutrient exchange and waste removal. Recent scrutiny of angiogenesis in female breast cancer has shown it to be of significant prognostic value. It was hypothesized that this holds true in invasive ductal carcinoma of the male breast. In the context of male breast cancer, we investigated the relationship of survival and other clinico-pathological variables to the microvascular density of the tumor tissue.

**Methods:**

Seventy-five cases of primary male breast cancer were identified using the records of the Saskatchewan Cancer Agency over a period of 26 years. Forty-seven cases of invasive ductal carcinoma of the male breast had formalin-fixed paraffin-embedded tissue blocks that were suitable for this study. All cases were reviewed. Immunohistochemical staining was performed for the angiogenic markers (cluster designations 31 (CD31), 34 (CD34) and 105 (CD105), von Willebrand factor (VWF), and vascular endothelial growth factor (VEGF)). Microvascular density (MVD) was determined using average, centre, and highest microvessel counts (AMC, CMC, and HMC, respectively). Statistical analyses compared differences in the distribution of survival times and times to relapse between levels of MVD, tumor size, node status and age at diagnosis. In addition, MVD values were compared within each marker, between each marker, and were also compared to clinico-pathological data.

**Results:**

Advanced age and tumor size were related to shorter survival times. There were no statistically significant differences in distributions of survival times and times to relapse between levels of MVD variables. There was no significant difference in MVD between levels of the different clinico-pathological variables. MVD was strongly and significantly correlated between AMC, CMC and HMC for CD31, CD34, and CD105 (p < 0.01) and remained moderate to weak for VWF and VEGF.

**Conclusion:**

Microvascular density does not appear to be an independent prognostic factor in male breast cancer. However, the likelihood of death for men with breast cancer is increased in the presence of increased age at diagnosis and advanced tumor size. This is perhaps linked to inherent tumor vasculature, which is strongly related throughout a tumor section.

## Background

Invasive ductal carcinoma of the male breast comprises approximately 1% of all breast cancers. Invasive ductal carcinoma of the male breast is distinct from invasive ductal carcinoma of the female breast in both presentation and immunophenotype. Male breast cancer generally presents in older patients and at a more advanced stage than its female counterpart [1-3]. In contrast to female breast cancers, ductal carcinoma *in situ *is quite rare in men [4,5]. Male breast cancers are also predominantly of the invasive ductal adenocarcinoma, not otherwise specified (NOS) type. Invasive ductal carcinoma of the male breast, despite being a high-grade tumor is more likely to express estrogen receptor and/or progesterone receptor and is less likely to over-express P53 or Erb-B2 when compared to invasive ductal carcinoma in the female breast [6,7]. The combination of a unique male hormonal environment, in addition to the unique immunophenotype, points to a distinct, non-p53-dependant, pathway of tumor progression in the male. Yet, despite these differences, it appears that the overall prognosis for male and female breast invasive ductal carcinomas are similar in age and stage-matched studies [1,8-10].

Angiogenesis is the growth and proliferation of blood vessels from existing vasculature. This process is quiescent in normal tissues and becomes active in rapidly growing tissues – including solid tumors. It has been shown that, in order to overcome tissue death by hypoxia, tumor growth beyond 1–2 mm^3 ^is dependant upon the formation of new vasculature [11]. Angiogenesis is, thus, an established step in solid tumor progression. This has been studied in many cancers including colorectal cancer [12] non-small cell lung cancer [13,14], hepatocelullar cancer [15], melanoma [16] prostate cancer [17], breast cancer [18-24] and bladder carcinoma [25].

Most assessments of angiogenesis in female breast carcinoma have shown it to be of significant prognostic value [18-22]. However, not all studies in this field have observed such important clinical correlations to MVD [23,24]. There are a variety of techniques used to evaluate angiogenesis and the variability between studies is probably related to the varying techniques employed in this process.

Invasive ductal carcinoma of the male breast appears to be a unique and biologically different carcinoma [1]; it is not simply the appearance of female invasive ductal carcinoma in a male breast. Due to the rarity of the disease large cohorts are not readily available, and there is only a limited pool of published data exploring various facets of this important disease. In one study of 26 men with breast cancer, elevated MVD was associated with advanced stage of disease and poor outcome [26]. Another Japanese study confirms that angiogenesis is part of tumor progression in male breast cancer [27].

In an attempt to further characterize this rare tumor, the aim of the current study was to evaluate angiogenesis in invasive ductal carcinoma of the male breast by the assessment of microvascular density in tumor samples. Specifically, we investigated three questions: (1) do survival times and times to relapse differ between levels of MVD, demographic, and clinico-pathological variables; (2) do MVD measures differ between levels of demographic and clinico-pathological variables, and finally; (3) are different measures of MVD correlated within a section of tumor tissue? This study is an extension of our established work on immunophenotypic characterization of male breast carcinoma in Saskatchewan [6].

## Patients and methods

### Patients

After obtaining appropriate ethics approval from the University of Saskatchewan Advisory Committee on Human Experimentation, all cases (n = 75) of invasive ductal male breast cancer diagnosed between 1975 and 1997 were selected from the records of the Saskatchewan Cancer Agency. Detailed chart review was performed for cases where paraffin-embedded tissue samples were available (n = 59). After the removal of all cases with inadequate tissue sample, tissue staining and chart data, there remained 47 cases.

### Clinical and pathological studies

Sections were cut from paraffin-embedded tissue samples. The sections were stained with hematoxylin and eosin (H & E). A detailed histopathological assessment was performed. Clinical features were recorded including age at diagnosis, date of birth, node status, tumor size, treatment method, date of relapse, and date of death.

Age at diagnosis, tumor size, node status, disease-free survival and overall survival were the clinical variables of interest in this study. Age at diagnosis was determined from the patient chart. Tumor size and node status were determined from the pathology report. Overall survival (number of years patient survived since the diagnosis of invasive breast carcinoma) and disease-free survival (number of consecutive years the patient was alive without breast cancer or other cancer relapse related to the breast carcinoma since the date of diagnosis) were calculated from the information gathered in the chart review. In cases where multiple tissue blocks were available, all H & E sections were examined in order to select a representative tissue block with a large area of invasive tumor and satisfactory tissue integrity.

### Microvessel density determination

MVD determination was modeled after the method described by Kato *et al*., [18] and Weidner *et al*., [28]. Immunohistochemical staining was performed for CD31, CD34, CD105, VWF and VEGF. Staining was carried out on a representative section by the avidin-biotin-peroxidase (ABC) technique after antigen retrieval using appropriate positive and negative controls in all cases. The source and dilution for each antibody are presented in table 1.

**Table 1:** Source and dilution of antibodies used in this study

**Antibody**	**Clone**	**Dilution**	**Source**	**Positive Control**	**Negative**
CD31	JC70A	1/20	Dako	Human Tonsil	All markers used patient tissue stained in the absence of primary antibody as negative control.
CD34	QBEnd10	1/20	Dako	Human Tonsil	
CD105	4G11	1/25	Novacastra	Human Tonsil	
VWF	F8/36	1/40	Signet	Human Tonsil	
VEGF	Polyclonal	1/20	Zymed	Human Colon Cancer CEA	

Brown-staining areas, whether single endothelial cells or clusters of endothelial cells, regardless of the absence/presence of a lumen were counted as individual microvessels. Vessels that had a thick muscular layer were excluded from the count. Cases were evaluated in a random order. Two observers using a double-headed light microscope simultaneously performed all counts for CD31, CD34, VWF and VEGF. A single experienced observer assessed CD105. Observers were blinded to all clinical and pathological data. Average, central and highest microvessel counts (AMC, CMC, and HMC, respectively) were performed.

**Figure 1:**
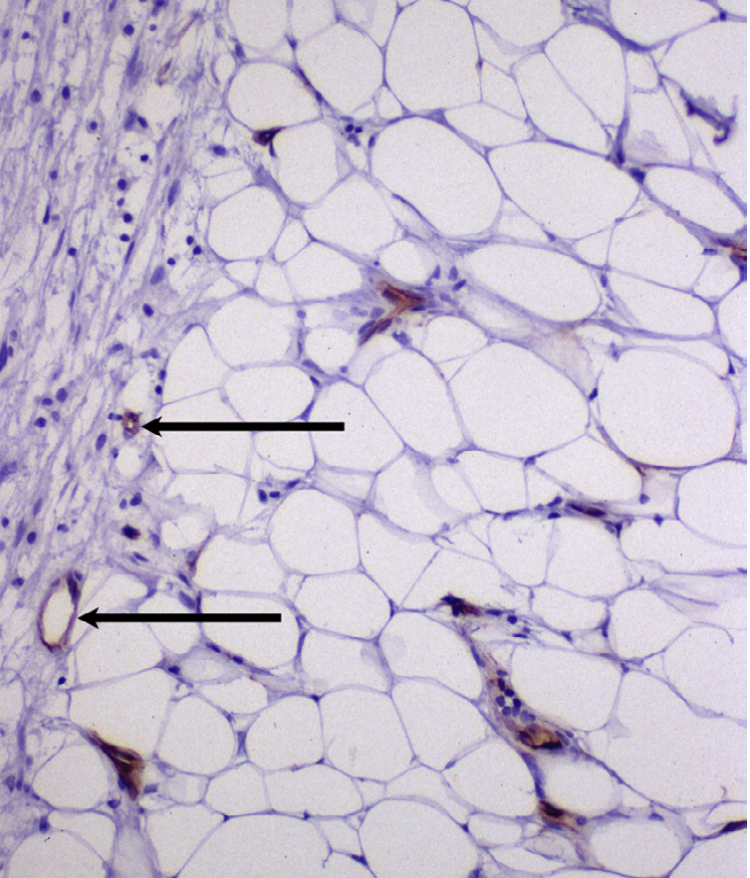
***Average microvessel count – VEGF. ***Ten high power (200×) fields along the border between cancer nests and the stroma were evaluated for each section. The average number of microvessels (arrows) per high power field was determined and reported as AMC.

Ten high power (200×) fields along the border between cancer nests and the stroma were evaluated for each section (figure 1). The average number of microvessels per high power field was determined and reported as AMC.

**Figure 2:**
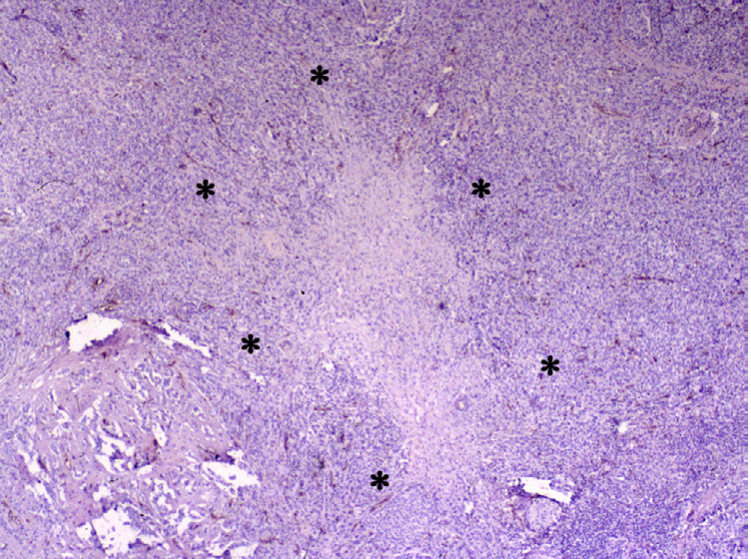
***Central microvessel count – VEGF. ***After scanning at low power (40×), the central area of the tumor was estimated. From this area, six high power (200×) fields were evaluated for each section. The average number of microvessels per high power field was determined and reported as CMC.

After scanning at low power (40×), the central area of the tumor was estimated. From this area, six high power (200×) fields were evaluated for each section (figure 2). The average number of microvessels per high power field was determined and reported as CMC. For tumors with a central necrotic area, determination was completed using areas near the centre of the tumor that were viable (non-necrotic).

**Figure 3:**
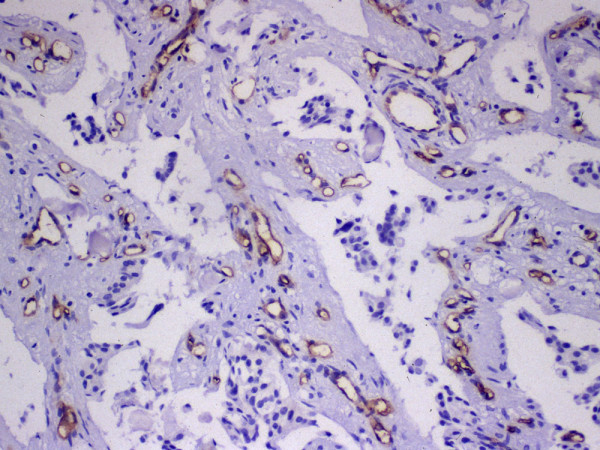
***Highest microvessel count – VEGF. ***After scanning at low power (40×), three areas with the highest concentration of microvessels (vascular hot spots) were selected. Each area was evaluated with one high power (200×) field in such a way as to include the maximum number of microvessels. The highest value obtained among the three fields was reported as HMC.

After scanning at low power (40×), three areas with the highest concentration of microvessels (vascular hot spots) were selected. Each area was evaluated with one high power (200×) field in such a way as to include the maximum number of microvessels (figure 3). The highest value obtained among the three fields was reported as HMC.

### Statistical analysis

Analysis was completed using the Statistical Package for the Social Sciences (SPSS) version 11.0 on an IBM PC 300PL computer. All tests were two tailed with the level of statistical significance set at p < 0.05. The demographic and clinico-pathological variables of interest included age at diagnosis (<65 and ≥ 65 years), tumor size (T1 is ≤ 2 cm, T2 is >2 cm but ≤ 5 cm, and T3 is >5 cm) and node status (positive and negative).

To compare the distribution of survival times and disease free survival times (time to relapse) we produced Kaplan-Meier curves and made statistical comparisons using the log-rank test between levels of demographic and clinico-pathological variables. In addition to this we dichotomized the MVD variables based on the median and repeated the Kaplan Meier with log-rank analysis to compare survival times and times to relapse between levels of MVD. For comparison of survival times, the outcome of interest was death while the remaining subjects (those surviving to the end of the study period) were censored. For comparison of time to relapse, the outcome of interest was relapse while the remaining subjects (those surviving to the end of the study or those who died before relapse) were censored.

Levels of MVD were also compared with levels of demographic and clinico-pathological variables using the Mann Whitney test or Kruskal Wallis test when MVD was considered as a continuous variable and chi squared or Fisher's Exact test when MVD was considered as dichotomous variable.

Finally, for each vascular marker (CD31, CD34, CD 105, VWF, and VEGF), correlation between the different measures of MVD (i.e. AMC with CMC, AMC with HMC, and CMC with HMC) was assessed using the Spearman's correlation coefficient. Correlations with a coefficient (ρ) of ≥ 0.80 were considered strong, moderate-strong correlations had coefficients that were <0.80 but ≥ 0.50, moderate-weak correlations had coefficients that were <0.50 but ≥ 0.30, weak correlations had coefficients that were <0.30.

## Results

### Age at diagnosis and clinicopathological characteristics

**Figure 4:**
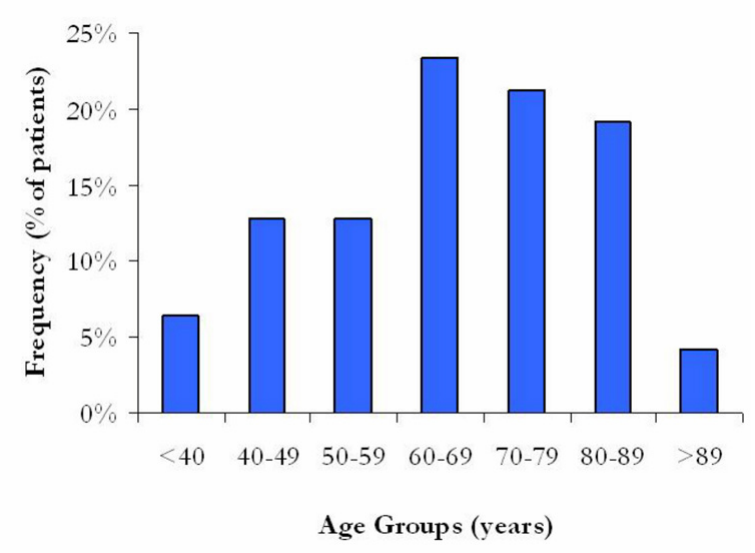
***Frequency of male breast cancer cases by age. ***This illustrates the age distribution of male breast cancer patients in this study. This is expressed as a percentage of the total number of patients. Note the predilection for older men.

In this study of 47 cases of male breast cancer, the median age of diagnosis was 65.9 years with the youngest being 32 years and the oldest being 94 years. The frequency of male breast cancer cases by age is illustrated in figure 4. As seen in Table 2, most of the patients had a tumor size of T1 to T2 and were node status negative.

**Table 2:** Clinico-pathological characteristics and survival of the study population. The adjacent table is a summary of clinico-pathological data of interest in this study.

**Characteristics**	**No. of cases**	**%**
		
Number of patients	47	100
		
Tumor size, T		
T1 (≤ 2 cm)	20	43
T2 (>2 cm, ≤ 5 cm)	22	47
T3 (>5 cm)	5	11
		
Node status		
N(-)	26	55
N(+)	21	45
		
Overall survival, years		
<10	27	64
≥ 10	15	36
Total evaluated	42	100
		
Relapse-free survival, years		
<10	29	69
≥ 10	13	31
Total evaluated	42	100

### Treatment regimens

All patients underwent some form of surgical resection – most frequently a modified radical mastectomy. In 31 out of 47 cases, surgical resection was followed by some form of adjuvant therapy (radiotherapy, chemotherapy, hormonal therapy (tamoxifen), or some combination of the aforementioned). Specifically, 6 patients received only radiotherapy, 7 patients received only hormonal therapy and 2 patients received only chemotherapy. For combined therapies, 6 patients received radiotherapy with hormonal therapy, 3 patients received radiotherapy with chemotherapy, 4 patients received hormonal therapy with chemotherapy and 3 patients received all three methods of adjuvant therapy.

### Patient outcome

All cases reviewed in this study came from the records of the Saskatchewan Cancer Agency between 1975 and 1997. Thirty-three of 47 patients (70%) died in the time period considered. Of the remaining 14 patients (30%), 9 (64%) had been followed for 10 years or more and 5 (36%) patients had been followed for under 10 years. Seventeen patients (36%) had documented relapse. The average age at death for patients with relapse was 72 years while the average age at death for relapse-free patients was 78 years. Although 70% of patients did die in this study, thirty-two patients (68%) survived at least 5-years after the diagnosis of breast cancer.

**Figure 5:**
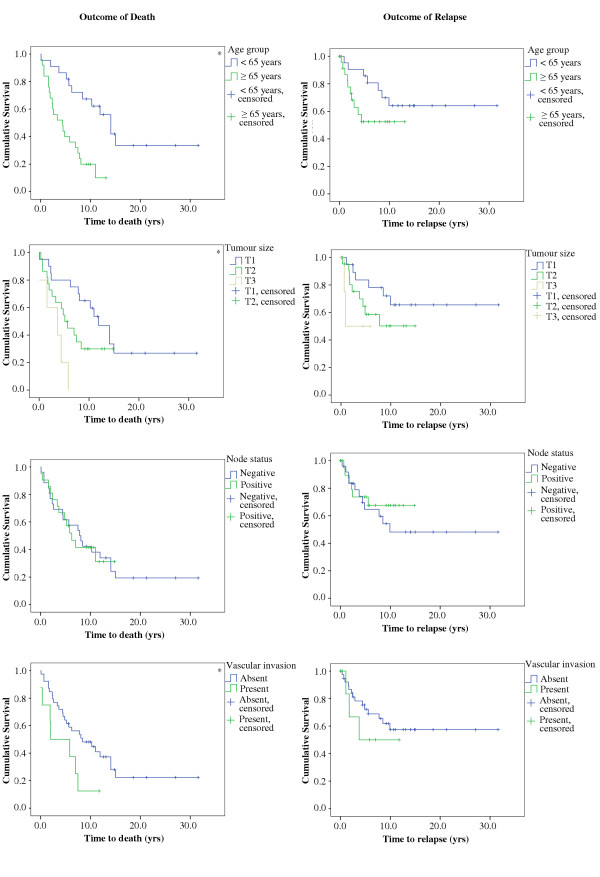
***Kaplan Meier curves for clinical variables with time to death (left column) and time to relapse (right column). ***This figure illustrates the percentage of patients with relapse-free survival across clinical groupings of age, node status and tumor size.

The Kaplan-Meier curves relating prognostic variables to death and relapse are illustrated in figure 5. There were significantly shorter survival times when the age of diagnosis was ≥ 65 years (p < 0.001) and when tumor size was larger (p < 0.01). However, there were no significant differences in the times to relapse by any of the clinical variables. In addition to this, there were no significant differences in survival times or times to relapse for any of the MVD markers when categorized by the median score (Table 3).

**Table 3:** Results from log rank test based on Kaplan Meier curves for microvascular density variables categorized at their median. The adjacent table reports the test statistic and p-value from the log rank test comparing differences in survival time and time to relapse between different levels of clinico-pathological variables.

	**Death**		**Relapse**	
		
	**Log rank test statistic**	**p-value**	**Log rank test statistic**	**p-value**
VWF				
AMC	0.04	0.83	0.46	0.50
CMC	0.70	0.40	0.02	0.88
HMC	0.01	0.93	0.45	0.50
CD31				
AMC	1.79	0.18	0.05	0.83
CMC	0.36	0.55	0.00	0.98
HMC	0.15	0.70	0.60	0.44
CD34				
AMC	0.19	0.67	0.91	0.34
CMC	0.19	0.66	0.37	0.54
HMC	0.08	0.78	0.30	0.59
CD105				
AMC	0.59	0.44	0.01	0.93
CMC	0.10	0.75	0.70	0.40
HMC	0.23	0.63	0.06	0.80
VEGF				
AMC	0.21	0.65	0.32	0.57
CMC	1.70	0.19	0.44	0.51
HMC	0.47	0.49	0.25	0.61

### Microvascular density and clinical variables

Regardless of whether MVD was considered as a continuous variable or as a categorical variable, there were no significant differences in MVD by demographic (age group) or clinico-pathological features (tumour size or node status) although some of the differences may be clinically important. Table 4 provides median values of MVD markers at different levels of clinico-pathological variables.

**Table 4:** Microvascular density levels at various levels of clinico-pathological variables. The adjacent table reports median MVD levels at different levels of clinico-pathological variables. None of the comparisons are statistically significant

	**Age group**				**Tumor size**						**Node status**			
	***<65 years***		***<65 years***		***T1***		***T2***		***T3***		***Negative***		***Positive***	
**VWF**														
AMC	15.6	(5.7)	15.0	(5.8)	15.8	(5.5)	13.5	(5.3)	17.5	(27.9)	14.2	(5.2)	15.6	(5.2)
CMC	53.7	(24.7)	47.2	(50.2)	44.8	(30.5)	50.3	(43.7)	67.5	(40.3)	48.5	(39.8)	50.0	(35.6)
HMC	84.0	(50.3)	70.0	(48.5)	86.5	(48.6)	69.0	(58.5)	70.0	(41.8)	84.0	(53.5)	69.3	(44.0)
**CD31**														
AMC	5.9	(20.9)	14.5	(28.8)	6.8	(28.6)	11.4	(30.5)	15.4	(61.7)	7.2	(17.9)	10.4	(32.6)
CMC	18.8	(41.4)	26.2	(28.6)	15.7	(38.0)	29.3	(28.9)	20.7	(81.0)	17.9	(33.7)	32.5	(36.5)
HMC	36.5	(44.8)	38.0	(27.0)	28.0	(40.0)	43.6	(22.3)	30.0	(107.4)	35.9	(40.0)	43.0	(42.6)
**CD34**														
AMC	19.7	(9.6)	19.8	(10.5)	21.0	(9.0)	17.3	(11.5)	20.0	(81.8)	18.8	(8.3)	19.9	(11.4)
CMC	52.0	(21.8)	41.3	(35.0)	43.3	(29.4)	52.0	(34.2)	48.5	(46.5)	47.2	(35.0)	50.7	(29.0)
HMC	89.0	(50.5)	70.0	(77.0)	79.0	(54.3)	86.0	(83.1)	88.0	(79.2)	90.0	(63.3)	70.0	(60.9)
**CD105**														
AMC	2.7	(4.4)	4.4	(4.3)	2.7	(3.8)	4.4	(5.4)	5.4	(10.0)	3.0	(4.7)	4.0	(3.6)
CMC	8.8	(14.3)	10.7	(13.2)	7.0	(13.8)	10.9	(14.9)	14.3	(31.7)	9.2	(14.5)	10.2	(10.6)
HMC	29.0	(24.5)	32.0	(34.5)	28.5	(30.3)	30.5	(49.3)	39.0	(156.0)	26.5	(33.0)	31.0	(20.0)
**VEGF**														
AMC	7.5	(20.0)	17.9	(25.1)	11.9	(25.5)	15.4	(26.8)	5.8	(64.9)	13.4	(21.8)	13.2	(29.2)
CMC	42.5	(47.0)	39.4	(33.6)	47.5	(72.9)	40.8	(39.5)	36.3	(45.6)	41.1	(45.1)	42.3	(58.8)
HMC	58.8	(103.1)	80.0	(87.6)	70.3	(113.6)	66.0	(79.1)	80.0	(55.9)	69.8	(86.2)	79.0	(92.8)

None of the differences between levels of age or clinico-pathological variables are statistically significant

### Microvascular density within individual markers

**Figure 6:**
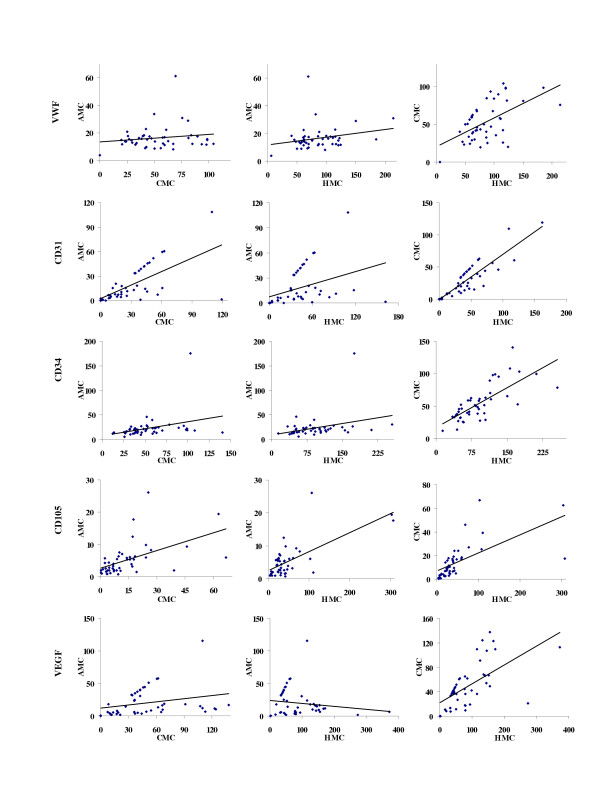
***Microvascular density correlations within each marker. ***This figure illustrates all the relationships between the different methods of MVD measurement for each marker.

Measures of MVD (AMC, CMC and HMC) were compared within each marker. These correlations are illustrated in figure 6.

#### CD31

Significant correlations (p < 0.01) were observed between all methods of measure (AMC correlates with CMC, CMC correlates with HMC, and HMC correlates with AMC) for CD31. The correlations between AMC and CMC, and AMC and HMC were moderate-strong (ρ = 0.76 and ρ = 0.60 respectively); the correlation between CMC and HMC was strong (ρ = 0.88).

#### CD34

Significant correlations (p < 0.01) were observed between all methods of measure (AMC correlates with CMC, CMC correlates with HMC, and HMC correlates with AMC) for CD34. All correlations were moderately-weak or moderate-strong (ρ = 0.45 for AMC and CMC, ρ = 0.41 for AMC and HMC, and ρ = 0.77 for CMC and HMC).

#### CD105

Significant correlations (p < 0.01) were observed between all methods of measure (AMC correlates with CMC, CMC correlates with HMC, and HMC correlates with AMC) for CD105. The correlations between AMC and CMC, and AMC and HMC were moderate-strong and moderate-weak (ρ = 0.62 and ρ = 0.49 respectively); the correlation between CMC and HMC was strong (ρ = 0.82).

#### VWF

A significant correlation (p < 0.01) was observed between CMC and HMC for VWF. A trend correlation (p < 0.10) was observed between AMC and HMC. Correlations were moderate-weak and weak (ρ = 0.47 and ρ = 0.25 respectively). There was no significant relationship between AMC and CMC for this marker.

#### VEGF

Significant correlations (p < 0.01) were observed between AMC and CMC, and between CMC and HMC for VEGF. Correlations were moderate-weak and moderate-strong (ρ = 0.43 and ρ = 0.68 respectively). There was no significant relationship between AMC and HMC for this marker.

## Discussion

### The markers

VEGF also called vascular permeability factor (VPF) is an important angiogenic activator, for both physiological and pathological angiogenesis [29,30], and it may be associated with inflammation. VEGF plays an essential role in embryonic vasculogenesis and angiogenesis [31,32]. It has also been implicated in postnatal development of the glomerulus [33,34] and endochondral bone [35,36].

VEGF mRNA has been shown to be up-regulated in the majority of human tumors investigated [37], and carcinoma of the human breast is one of these. [38,39]. In addition, VEGF has been implicated in psoriasis [40], brain edema [41], polycystic ovary syndrome [29], age-related macular degeneration (AMD) and other intraocular neovascular syndromes [42-44] The expression of VEGF is triggered by hypoxia. That is to say, low oxygen tension provokes VEGF mRNA expression [45].

An excellent review of CD105 and its involvement in angiogenesis has been written by Duff *et al*., [46]. CD105 (endoglin) is commonly expressed by angiogenic endothelial cells [46-48]. CD105 is an important pro-angiogenic factor. Transforming growth factor β exerts an inhibitory influence on cell proliferation, migration and microvessel formation. The suppressive effect of CD105 on transforming growth factor β, thus, contributes to angiogenesis [49]. It is, therefore, no surprise to observe elevated CD105 expression in various tumor endothelia [50-52], including breast cancer [53]. CD105 may be shed into the blood stream. The measure of serum endoglin appears to provide important prognostic information in cancer patients [54,55].

CD31 is an important part of the endothelial intercellular junction [56] and it plays a crucial role in leukocyte migration through vascular endothelial intracellular junctions [57-59]. This molecule is at least partially responsible for the adhesion between leucocytes/endothelial cells, leucocytes/platelets, and endothelial cells/endothelial cells [57,60-65]. This adhesion is likely the result of CD31-CD31 [66] interactions (homophilic interactions) although adhesion between CD31 and other components of the cell membrane has been demonstrated (heterophilic interactions) [61,67-70].

CD31 also exhibits signal transduction; its dimerization appears to upregulate integrin function [71]. This molecule appears to be involved in thrombosis, angiogenesis, wound healing, and inflammation [61]. CD31 is known to be a co-signal transducer for macrophages, inducing respiratory burst.

CD34 is a glycosylated type I transmembrane protein [72] which is expressed on hematopoietic stem cells, committed hematological progenitor cells [73-75], small vessel endothelial cells [76,77], tumors of epithelial origin [78,79] and a limited number of other cell populations including some haematological malignancies [72].

As specific ligands are still undefined, the precise role CD34 plays in early hematopoiesis remains uncertain. It is thought that differential splicing of sugar residues on CD34 may permit it to host a variety of ligands under different conditions [80]. Despite our meager understanding of this complex molecule there is evidence indicating that hematopoietic CD34 plays a role in modulating adhesion (this has been reviewed previously [72]).

Factor VIII related antigen, or von Willebrand factor (VWF), is a plasma protein produced by endothelial cells [81,82]. VWF is also present in platelets, as it is produced by their megakaryocytic precursor [83].

VWF is a multifunctional protein. It is known to mediate adhesion/aggregation of platelets in clot formation (reviewed in [84]). In addition to this, VWF acts as a chaperone for circulating factor VIII. About 1 – 2% of VWF is bound by factor VIII [85]. This non-covalent bond prolongs the survival of factor VIII in the plasma. When the coagulation cascade is triggered, thrombin cleaves the complex, thereby freeing factor VIII to participate coagulation [86] (reviewed in [87]).

### Age at diagnosis

Male breast cancer is a disease of older men. The likelihood of this occurring in older men that is illustrated in this study is not surprising as this is the case for most studies of e breast cancer in males [88,89]. As mortality from common conditions (e.g. cardiovascular disease) within this group improves due to advances in treatment/intervention and a larger proportion of the population enters this age group, it seems that the relative incidence of male breast cancer is likely to rise. Such is the finding in a recent meta-analysis of male breast carcinoma [1].

### Survival

In this study, 70% of the reviewed patients died. Though this number may seem high, only half of those who died had documented relapse prior to the time of death. There is, however, an interesting difference between average age at death for relapsed and relapse-free patients, 72 years versus 78 years respectively. It appears that male breast cancer is contributing to mortality, but this study did not examine the effects of co-morbid conditions. The expected life remaining for a 65 year old male in Saskatchewan between 1995 and 1997 was 16.7 years (expected age approximately 82 years) [90].

Increased tumor size increases the likelihood of death for male breast cancer patients in this study (figure 5). One possible explanation for this relationship is as follows: a tumor's size may be a function of its rate of growth and time of growth; these characteristics seem likely to increase the opportunity for relapse and metastasis. Thus, we might expect large tumors to relapse more frequently than small ones, and therefore, also contribute to death.

It appears that younger patients had a significantly better chance of not experiencing death (figures 5). This phenomenon is possibly related to improved response to treatment in younger patients; alternatively, this relationship may be demonstrating that younger patients are diagnosed with less advanced disease and vice versa. Evidence supports advanced age [88,89] and tumor size [91] as important negative prognostic factors.

This study was not able to clearly demonstrate statistically significant differences in survival for node status. In the available literature axillary node status is an important prognostic factor [91-94].

Microvascular density, though it was the primary focus of this study, did not demonstrate statistically significant association with survival, demographic or clinico-pathological features. However, we cannot discount the importance of angiogenesis in tumor progression. The lack of correlation in this study may have been influenced by the lack of statistical power, the methods used, the age of the tissue, advanced stage of disease at presentation and method of analysis.

In most tumors studied, MVD has been identified as a prognostic factor and has had important correlations to clinical variables [12-16]. In most studies where angiogenesis has been evaluated in cancer of the female breast, MVD is an important prognostic factor [19-22]. In one study of male breast cancer using CD34 to highlight vessels, it was concluded that MVD was an important prognostic tool [26].

In an angiogenesis methodology study of 109 women with breast cancer by Kato *et al*., [18] it was found that CMC and HMC did not correlate to clinico-pathological variables other than peritumor vascular invasion. AMC was found to have prognostic value. The methods used to report microvessel density were modeled after this work by Kato *et al*, [18].

Despite a lack of strong evidence, in our study, to support angiogenesis as an independent prognostic factor, there is no evidence to disprove angiogenesis plays a critical role in tumor development. As angiogenesis remains a likely step in tumor progression, we must continue to recognize this process as a potential target for anti-tumor therapy.

### Microvascular density within each marker

There were some important correlations between the different methods of measure for MVD (AMC, CMC and HMC) within the various markers. CD31, CD34 and CD105 were the strongest in this regard with correlations that were very significant (p < 0.01) and correlations that were usually moderate to strong. The correlations within VWF and VEGF were not all significant, and the relationship was moderate to weak.

It could also be that VWF and VEGF are differentially expressed in male breast cancer tissue. This seems to be the case for VEGF. In fact, it was observed that VEGF had a propensity to be over-expressed in regions where there were invading lymphocytes. This may produce a patchy pattern of expression, which could have an important effect on microvessel counts.

For the most part, this study saw strong correlations between the various microvessel count methods within the markers. Critics may suggest that evaluation of microvascular density for prognosis in tumors is flawed because, within a tumor, microvascular density is heterogeneous [24,95]. However, the correlations observed in this study support the notion that tumor vasculature is predictable (but not ubiquitous or necessarily homogeneous) from the centre, periphery and vascular hotspot of a tumor. Notably, similar research in female invasive ductal carcinoma of the breast using VWF MVD assessment techniques also demonstrated correlation between central, peripheral and highest microvessel densities [18].

Microvessel determination, by the methods used in this study, is dependant on a predictable pattern of vasculature within a tumor. Such predictability allows for practical (in terms of time, money and ease of use) application of important clinical prognostic features of the markers. Further research to examine the relationship between these markers in cancer is wanting. Such information may prove important in improving the prognostic value of MVD determination.

## Conclusion

From this evaluation of angiogenesis in male breast cancer, we can draw the following conclusions:

Microvascular density does not appear to be an independent prognostic factor in male breast cancer. Tumor vasculature (as measured by microvessel determination using antibodies to endothelial markers such as CD31, CD34, CD105) is strongly related throughout a tumor section (p < 0.01). Other endothelial markers such as VWF and VEGF appear to have a moderate to weak relationship. Advanced age at diagnosis and increased tumor size increases the likelihood of death for men with breast cancer.

## Abbreviations

AMC Average microvessel count

CD# Cluster designation or cluster of differentiation (CD31, CD34, CD105)

CMC Central microvessel count

HMC Highest microvessel count

MVD Microvessel density

TNM Tumour nodes metastasis

VEGF Vascular endothelial growth factor

VWF Von Willebrand factor

## Competing interests

The authors declare that they have no competing interests.

## Authors' contributions

**EF **wrote this manuscript, aided in collection and analysis of data and is the first author.

**JL **provided statistical analysis of the collected data.

**RK **conceived the design of this study, aided in data collection and remains the corresponding and senior author.

All authors have read and approved this manuscript.
